# Supplementary material for: The Structure of the “Vibration Hole” around an Isotopic Substitution—Implications for the Calculation of Nuclear Magnetic Resonance (NMR) Isotopic Shifts
Source: Molecules. 2020 Jun 24;25(12):2915. doi: 10.3390/molecules25122915 (PMC7355873; doi:10.3390/molecules25122915)
Supplement: Supplementary file 1 [file molecules-25-02915-s001.zip › Grafenstein-Molecules-SI-rev1/Grafenstein-Molecules-SI-text-rev1.pdf]

Supporting information

Jürgen Gräfenstein

The structure of the ”vibration hole” around an isotopic substitution. Implications for the calculation of NMR isotopic shifts

Contents

S1 Description of the zip file in the SI **S1**

S2 Isotope effects on geometry parameters and NMR isotopic shifts **S2**
S2.1 Molecules in Sec. 3.1 . . . . S2
S2.2 Molecules in Sec. 3.2 . . . . S7
S2.3 Molecules in Sec. 3.3 . . . . S9

List of Tables

S1 NMR isotopic shifts for a single H/D substitution in benzene S2
S2 NMR isotopic shifts for a single H/D substitution in cyclohexane S2
S3 NMR isotopic shifts for a single H/D substitution in norbornane S3
S4 NMR isotopic shifts for a single H/D substitution in adamantane S3
S5 Isotope effects on selected bond distances in the compounds considered in Sec. 3.1 . . . . S4
S6 Isotope effects on selected mean-square vibration amplitudes in the compounds considered in Sec. 3.1 . . . . S5
S7 Isotope effects on the amplitude covariances with in the compounds considered in Sec. 3.1. . . . . S6
S8 NMR isotopic shifts for a H/D substitution at H2 in pyridine and bpeb-derivative complexes S7
S9 Isotope effects on selected bond distances in pyridine and bpeb-derivative complexes S7
S10 Isotope effects on the mean-square vibration amplitudes in pyridine and bpeb-derivative complexes S8
S11 Isotope effects on the amplitude covariances with in pyridine and bpeb-derivative complexes S8
S12 Isotope effects on selected bond distances and mean-square vibration amplitudes in pyridine and **bpeb**-derivative complexes S8
S13 NMR isotopic shifts, r_e values for and isotope effects on selected bond distances in **sal** and its derivatives S9
S14 Isotope effects on selected mean-square amplitudes in **sal** and its derivatives S10
S15 Isotope effects on selected amplitude covariances with $r(\mathrm{O\underline{H}})$ in **sal** and its derivatives S11
S16 Equilibrium values for and isotope effects on selected bond angles in **sal** and its derivatives S12

S1 Description of the zip file in the SI

The zip archive contains three kinds of ASCII files:

1. one text file (suffix .txt) for each compound investigated in this work. The names of these files reflect the compound under consideration.

   The .txt file contains a description of the computational models for each calculation of the force field and the NMR properties. Additionally, for each force-field calculation, the Cartesian geometry (all coordinates in Å), the ground-state energy (in Hartree) and the zero-point vibration energy (in kJ/mol) are given. For each calculation of NMR properties, the corrsponding ground-state energy is given.

2. one file (suffix .xyz) for each force-field calculation with the corresponding equilibrium geometry in xyz format. The names of these files reflect the compound in question; for pyridine in addition the basis set and solvent.

3. one file (suffix .molden) for each force-field calculation with the corresponding difference-dedicated vibration modes in Molden [S1] format. Instead of the vibration frequencies, the weight factors κ_i are given, all intensities are put to one.

S2 Isotope effects on geometry parameters and NMR isotopic shifts

S2.1 Molecules in Sec. 3.1

Table S1: NMR isotopic shifts for a single H/D substitution in benzene calculated at various levels of theory. Calculations done with ωB97X-D/pc-2 force field. All values calculated for acetone solution and given in ppb.

method	basis set	C1	C2	C3	C4	MSgD	RMS
DD-VPT2 [S2]	ωB97X-D	−317.9	−141.7	−9.9	−5.6	−21.5	26.9
	B3LYP	−355.2	−147.0	−11.9	−3.4	−36.4	46.6
loc-VPT2	ωB97X-D	−282.3	−135.2	−8.7	−19.5	−7.1	14.0
	B3LYP	−320.8	−140.5	−10.4	−17.3	−22.2	27.7
LMZL	ωB97X-D	−230.5	−130.1	−8.2	−21.4	12.1	32.3
	B3LYP	−243.0	−137.8	−9.5	−18.4	4.9	27.8
LMZL+cent	ωB97X-D	−273.9	−130.8	−8.7	−20.2	−2.8	12.7
	B3LYP	−293.0	−138.9	−10.0	−17.2	−12.3	17.1
Experiment [S3]		−283	−111	−11			

Table S2: NMR isotopic shifts for a single H/D substitution in cyclohexane calculated at various levels of theory. Calculations done with the ωB97X-D XC functional and a pc-2 basis set for the force fields and a pcS-2 basis set for the NMR calculations. All values given in ppb.

		C1	C2	C3	C4	H2		MSgD	RMS
method	basis set					ax	eq		
H/D substitution at axial site									
DD-VPT2 [S2]	ωB97X-D	−494.4	−102.4	−12.0	1.4	−6.5	−7.4	−17.8	30.3
	B3LYP	−525.6	−105.4	−8.4	3.0	−7.0	−7.6	−28.0	48.4
loc-VPT2	ωB97X-D	−510.9	−99.3	−20.9	0.2	−5.6	−7.1	−25.2	39.8
	B3LYP	−544.2	−102.9	−17.2	1.5	−6.1	−7.3	−36.2	58.9
LMZL	ωB97X-D	−399.1	−90.6	−16.4	0.8	−5.4	−6.1	16.5	25.3
	B3LYP	−429.8	−96.6	−13.1	1.5	−5.8	−6.4	5.3	7.3
	B3LYP [S4]	−403.7	−88.6	−14.3	−3.0	−5.6	−6.4	16.3	23.0
LMZL+cent	ωB97X-D	−500.8	−100.4	−20.0	0.7	−5.7	−6.9	−21.9	34.0
	B3LYP	−537.0	−106.8	−16.3	1.4	−6.1	−7.3	−34.9	54.9
experiment	Ref. [S5]	444.9							
	Ref. [S6]					−6.9	−7.5		
(t-Bu-C_6H_{12})	Ref. [S5]	442.2	98.6	14.7					
H/D substitution at equatorial site									
DD-VPT2 [S2]	ωB97X-D	−439.6	−114.7	−40.0	0.2	−11.2	−7.9	−19.3	27.7
	B3LYP	−478.8	−123.6	−43.6	−0.2	−11.5	−8.1	−36.5	51.0
loc-VPT2	ωB97X-D	−439.4	−118.5	−30.3	−0.1	−11.6	−8.4	−17.3	28.4
	B3LYP	−480.3	−128.0	−33.6	−0.8	−12.0	−8.6	−35.2	52.4
LMZL	ωB97X-D	−327.9	−105.9	−29.3	−0.4	−9.8	−7.1	24.4	37.6
	B3LYP	−373.2	−115.2	−33.0	−1.3	−10.0	−7.3	5.0	12.5
	B3LYP [S4]	−354.2	−114.7	−27.1	−2.6	−9.9	−8.0	13.4	23.4
LMZL+cent	ωB97X-D	−418.3	−116.3	−30.8	0.0	−11.2	−8.0	−9.7	16.5
	B3LYP	−470.2	−126.4	−34.7	−1.0	−11.4	−8.2	−31.7	46.4
experiment	Ref. [S5]	396.4							
	Ref. [S6]					−10.4	−7.5		
(t-Bu-C_6H_{12})	Ref. [S5]	392.4	106.2	37.7					

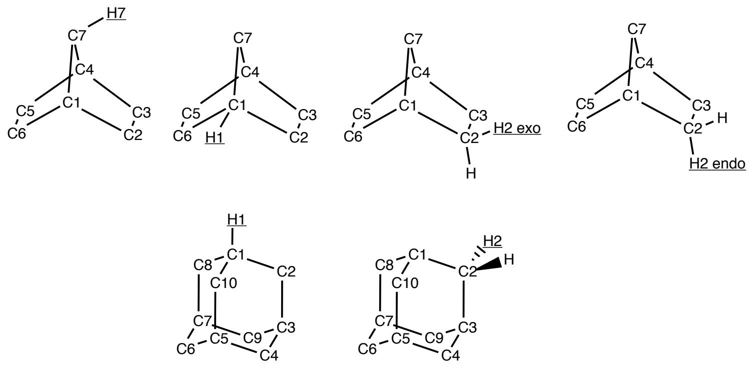

Figure S1: Atom numbering for norbornane and adamantane. The H/D substitution sites are underlined.

Table S3: NMR isotopic shifts for a single H/D substitution in norbornane calculated at various levels of theory. Calculations done with the ωB97X-D XC functional and a pc-2 basis set for the force fields and a pcS-2 basis set for the NMR calculations. DD-VPT2 values from Ref. [S2], experimental values from Ref. [S7]. All values given in ppb.

substitution	method/ condition	C1	C2	C3	C4	C5	C6	C7
H7	DD-VPT2	−100.6	−31.9	−31.9	−100.6	2.0	2.0	−416.0
	loc-VPT2	−91.2	−30.8	−30.8	−91.2	14.5	14.5	−404.1
	expt (25°C)	−88.5	−26.2	−26.2	−88.5	6.6	6.6	−361.1
H1	DD-VPT2	−442.9	−119.6	−8.4	40.6	-8.4	−119.6	−108.4
	loc-VPT2	−423.5	−111.2	−7.7	39.5	-7.7	−111.2	−104.9
	expt (25°C)	−376.4	−103.6	−7.3	36.7	-7.3	−103.6	−91.2
H2 exo	DD-VPT2	−97.9	−418.1	−135.6	−12.9	7.0	−54.2	−18.0
	loc-VPT2	−95.5	−399.9	−117.6	−11.9	-3.3	−28.0	−19.1
	expt (25°C)	−87.4	−364.9	−120.2	−14.6		−43.7	−12.3
H2 endo	DD-VPT2	−111.8	−442.2	−100.4	10.6	-2.2	−52.1	−2.2
	loc-VPT2	−94.7	−434.7	−98.8	9.5	-0.7	−82.3	17.1
	expt (25°C)	−94.3	−384.0	−89.1	6.7		−50.8	5.2

Table S4: NMR isotopic shifts for a single H/D substitution in adamantane calculated at various levels of theory. Calculations done with the ωB97X-D XC functional and a pc-2 basis set for the force fields and a pcS-2 basis set for the NMR calculations. DD-VPT2 values from Ref. [S2], experimental values from Ref. [S8]. All values given in ppb.

substitution	method/ condition	C1	C2	C4	C5	C6	C7	C8
H1	DD-VPT2	−595.9	−140.8	2.2	-32.7	2.2	−32.7	−140.9
	loc-VPT2	−596.9	−139.7	−1.2	-28.0	−1.2	−28.0	−139.6
	expt (25°C)	−514	−127		-32		−32	−127
H2	DD-VPT2	−105.9	−500.4	−13.5	0.5	−3.7	−9.1	−41.3
	loc-VPT2	−100.9	−510.9	−27.3	-4.0	−1.5	−5.1	−18.5
	expt (25°C)	−100	−440	−13		−4	−3	−31

Table S5: Isotope effects on selected bond distances in the compounds considered in Sec. 3.1. Calculations done at the ωB97X-D/pc-2 level of theory. The notations s, g, and a indicate bonds in syn, gauche, and anti conformation to the C–H bond, respectively. All values given in μÅ.

	r(C<u>H</u>)		r(CC′)		r(CH′)		r(C′C″)			r(C′H″)		
	r_z	r_g	r_z	r_g	r_z	r_g	r_z	r_g		r_z	r_g	
**Pyridine**	−989	−5437	−147	−182			−32	4	a	−1	3	s
**Benzene (H/D)**	−769	−5369	−140	−168			−30	2	a	7	2	s
**Cyclohexane**												
axial	−1740	−5987	−85	−147	338	15	11	20	g	17	20	g
										−31	−40	a
equatorial	−1563	−5851	−121	−182	361	18	−82	−54	a	4	17	g
										21	21	g
**Norbornane**												
H7	−1582	−5783	−166	−198	197	−5	12	18	g	7	12	g
							−63	−51	a			
H1	−1825	−5793	−169	−181			−16	−5	a	7	10	g
			−191	−207			−30	−11	a	2	8	g
										10	16	g
H2 exo	−1433	−5777	−104	−167	365	2	−75	−59	a	6	7	g
			−95	−184			28	24	g	23	−7	g
							−32	−10	g	47	19	s
H2 endo	−1424	−5761	−187	−251	376	12	19	31	g	13	18	g
			−72	−157			−49	−45	a	39	20	s
							−15	14	g	13	−12	g
**Adamantane**												
H1	−2396	−6130	−146	−161			−59	−54	a	13	14	g
H2	−1877	−6002	−137	−173	255	10	8	8	g	18	19	g
							−90	−86	a			
**Methane**	−1920	−5704			−855	103						

Table S6: Isotope effects on selected mean-square vibration amplitudes in the compounds considered in Sec. 3.1. Calculations done at the ωB97X-D/pc-2 level of theory. The letters s, g, and a indicate bonds in syn, gauche, and anti conformation, respectively, to the C–H̲ bond. All values given in mÅ^2 or dgr^2, respectively.

	$r(\text{C}\underline{\text{H}})$	$r(\text{CC}')$	$r(\text{CH}')$	$r(\text{C}'\text{C}'')$		$r(\text{C}'\text{H}'')$		φ_{az}	φ_{alt}
**Pyridine**	−1525	−4.17		−0.85	a	−0.0073	s	−9.8	−12.9
**Benzene**	−1512	−3.86		−0.90	a	−0.0058	s	−10.3	−13.6
**Cyclohexane**									
axial	−1584	−2.87	−0.267	−0.03	g	−0.0079	g	−10.5	−11.5
						−0.0136	a		
equatorial	−1567	−3.49	−0.363	−2.52	a	−0.0093	g	−10.7	−11.5
						−0.0187	g		
**Norbornane**									
H7	−1563	−4.70	−0.306	−0.17	g	−0.0060	g	−11.4	−11.3
				−1.76	a				
H1	−1553	−4.90		−1.58	a	−0.0086	g	−10.9	−11.2
		−4.52		−1.78	a	−0.0073	g		
						−0.0172	g		
H2 exo	−1560	−3.86	−0.343	−1.49	a	−0.0029	g	−11.0	−11.5
		−4.26		−0.26	g	−0.0036	g		
				−0.66	g	−0.0125	s		
H2 endo	−1563	−4.43	−0.272	−0.03	g	−0.0160	g	−10.8	−11.7
		−3.30		−2.38	a	−0.0117	s		
				−0.66	g	−0.0038	g		
**Adamantane**									
H1	−1579	−3.22		−2.68	a	−0.0120	g	−10.6	−10.4
H2	−1578	−3.72	−0.290	−0.08	g	−0.0106	g	−10.7	−11.4
				−2.11	a				

Table S7: Isotope effects on the amplitude covariances with $r(\mathrm{C\underline{H}})$ in the compounds considered in Sec. 3.1. Calculations done at the ωB97X-D/pc-2 level of theory. The letters s, g, and a indicate bonds in syn, gauche, and anti conformation, respectively, to the C–$\underline{\mathrm{H}}$ bond. All values given in mÅ^2 or dgr^2, respectively.

	$r(\mathrm{CC'})$	$r(\mathrm{CH'})$	$r(\mathrm{C'C''})$		$r(\mathrm{C'H''})$	
**Pyridine**	−35.8		−7.18	a	0.17	s
**Benzene**	−34.7		−7.44	a	0.44	s
**Cyclohexane**						
axial	−24.3	−5.91	0.78	g	1.14	g
					−1.93	a
equatorial	−27.4	−6.45	−7.25	a	1.03	g
					1.31	g
**Norbornane**						
H1	−31.2	−8.05	1.62	g	0.62	g
			−5.14	a		
H7	−33.7		−3.63	a	0.45	g
	−32.5		−4.64	a	0.33	g
					1.29	g
H2 exo	−28.8	−6.82	−5.48	a	0.31	g
	−31.4		1.82	g	−1.09	g
			−2.44	g	2.13	s
H2 endo	−27.8	−6.85	0.12	g	1.32	g
	−29.0		−6.99	a	2.57	s
			−3.66	g	−1.13	g
**Adamantane**						
H1	−26.2		−7.40	a	1.05	g
H2	−26.8	−6.62	1.43	g	1.16	g
			−7.51	a		

S2.2 Molecules in Sec. 3.2

Table S8: NMR isotopic shifts for a H/D substitution at H2 in pyridine and **bpeb**-derivative complexes calculated at various levels of theory. See Sec. 4 of the main text for computational details. All values given in ppb.

method/ condition	basis set	C2	C3	C4	C5	C6
**Pyridine**	DD-VPT2	−386.1	−168.4	3.2	20.7	−8.4
	loc-VPT2	−366.1	−164.2	4.1	2.8	−19.4
	expt (25°C)	−341.3	−140.4		14.1	−14.5
	expt (0 K)	−367.1	−166.4		24.2	−26.0
**bpeb-1a**	DD-VPT2	−377.7	−170.6	25.9	10.3	−32.1
	loc-VPT2	−338.7	−159.7	20.6	−3.4	−26.9
	expt (25°C)	−334.2	−144.9	23.7		−27.8
	expt (0 K)	−369.7	−175.4	13.8		−40.5
**bpeb-1b**	DD-VPT2	−375.9	−166.4	16.3	9.4	−21.1
	loc-VPT2	−333.4	−153.1	19.0	−5.0	−24.2
	expt (25°C)	−328.4	−140.7	21.9		−27.0
	expt (0 K)	−371.5	−184.5	26.6		−36.9
**bpeb-1c**	DD-VPT2	−358.0	−167.1	26.1	4.0	−24.2
	loc-VPT2	−326.3	−153.2	24.5	−4.7	−16.1
	expt (25°C)	−322.1	−145.2	21.4		−20.3
	expt (0 K)	−342.9	−190.4	30.3		−32.3
**bpeb-1d**	DD-VPT2	−353.5	−160.1	17.1	12.7	−21.6
	loc-VPT2	−323.7	−144.7	20.5	−3.8	−14.1
	expt (25°C)	−322.4	−143.4	15.6		−18.1
	expt (0 K)	−344.3	−183.0	28.5		0.6
**bpeb-2**	DD-VPT2	−339.3	−173.3	23.5	−6.9	−39.0
	loc-VPT2	−313.1	−166.7	17.2	−7.5	−25.0
	expt (25°C)	−315.3	−138.4	23.2		−24.8
	expt (0 K)	−343.6	−177.7	11.2		−43.3

Table S9: Isotope effects on selected bond distances in pyridine and **bpeb**-derivative complexes calculated at various levels of theory. See Sec. 4 of the main text for computational details. All values given in μÅ.

	r(C<u>H</u>)		r(CC′)		r(CN′)		r(C′C″)		r(C′H″)		r(N′C″)		r(N′X″)	
	r_z	r_g	r_z	r_g	r_z	r_g	r_z	r_g	r_z	r_g	r_z	r_g	r_z	r_g
**Pyridine**	−1126	−5558	−143	−177	−44	−74	−39	−3	−9	−5	−17	1		
**bpeb-1a**	−1144	−5571	−164	−206	−129	−181	66	23	23	0	0	−31	−44	−54
**bpeb-1b**	−1126	−5540	−174	−211	−104	−154	53	17	28	1	−11	−39	373	363
**bpeb-1c**	−1131	−5521	−126	−162	−120	−169	22	−13	49	16	−11	−38	366	357
**bpeb-1d**	−1201	−5586	−103	−138	−138	−184	−25	−51	45	7	7	−13	274	265
**bpeb-2**	−1173	−5580	−122	−162	−117	−171	19	−26	14	−10	21	−10	−174	−181

Table S10: Isotope effects on the mean-square vibration amplitudes in pyridine and **bpeb**-derivative complexes. See Sec. 4 of the main text for computational details. All values given in mÅ^2 or dgr^2, respectively.

	r(C$\underline{\text{H}}$)	r(CC′)	r(CN′)	r(C′C″)	r(C′H″)	r(N′C″)	r(N′X″)
**Pyridine**	−1517	−4.22	−1.94	−0.85	−0.00489	−0.83	
**bpeb-1a**	−1497	−4.03	−2.67	−0.82	−0.00313	−1.17	−2.94
**bpeb-1b**	−1498	−4.23	−2.70	−1.11	−0.00335	−1.16	−2.09
**bpeb-1c**	−1498	−4.12	−2.70	−1.20	−0.00483	−1.18	−1.72
**bpeb-1d**	−1500	−3.93	−2.84	−1.52	−0.00503	−1.16	−1.40
**bpeb-2**	−1496	−4.09	−2.71	−0.80	−0.00401	−1.15	−2.98

Table S11: Isotope effects on the amplitude covariances with r(C$\underline{\text{H}}$) in pyridine and **bpeb**-derivative complexes calculated at various levels of theory. See Sec. 4 of the main text for computational details. All values given in mÅ^2 or dgr^2, respectively.

	r(CC′)	r(CN′)	r(C′C″)	r(C′H″)	r(N′C″)	r(N′X″)
**Pyridine**	−35.8	−26.5	−7.22	−0.136	−6.14	
**bpeb-1a**	−36.5	−29.8	−6.92	−0.271	−7.13	3.98
**bpeb-1b**	−36.4	−29.9	−7.74	−0.238	−6.95	3.61
**bpeb-1c**	−35.5	−29.7	−7.95	−0.169	−6.99	3.49
**bpeb-1d**	−34.6	−30.0	−8.89	−0.064	−6.76	3.32
**bpeb-2**	−36.8	−29.5	−6.91	−0.354	−7.21	4.42

Table S12: Isotope effects on selected bond distances and mean-square vibration amplitudes in pyridine and **bpeb**-derivative complexes calculated at various levels of theory. See Sec. 4 of the main text for computational details. All values given in mÅ^2 or dgr^2, respectively.

		Geometry		Amplitude (mean square)					
		r(C$\underline{\text{H}}$)		r(C$\underline{\text{H}}$)		φ_{az}		φ_{alt}	
		r_z	r_g	all-H	IE	all-H	IE	all-H	IE
**Pyridine**	DD-VPT2	−1126	−5558	5671	−1517	38.7	−9.7	83.5	−12.8
	loc-VPT2	−1499	−5850	5440	−1592	35.3	−10.3	54.3	−15.9
**bpeb-1a**	DD-VPT2	−1144	−5571	5589	−1497	39.1	−9.8	82.8	−13.0
	loc-VPT2	−1467	−5842	5367	−1570	35.5	−10.4	54.8	−16.0
**bpeb-1b**	DD-VPT2	−1126	−5540	5592	−1498	39.0	−9.8	82.8	−13.0
	loc-VPT2	−1439	−5812	5369	−1571	35.5	−10.4	54.8	−16.0
**bpeb-1c**	DD-VPT2	−1131	−5521	5592	−1498	38.8	−9.7	83.1	−12.8
	loc-VPT2	−1453	−5801	5370	−1571	35.3	−10.3	54.5	−15.9
**bpeb-1d**	DD-VPT2	−1201	−5586	5603	−1500	38.8	−9.7	83.9	−12.8
	loc-VPT2	−1528	−5873	5380	−1574	35.3	−10.3	54.4	−15.9
**bpeb-2**	DD-VPT2	−1173	−5580	5587	−1496	39.2	−9.8	82.4	−12.9
	loc-VPT2	−1497	−5861	5364	−1570	35.7	−10.4	54.4	−15.9

S2.3 Molecules in Sec. 3.3

Table S13: NMR isotopic shifts $^2\Delta$(C2), equilibrium values for and isotope effects on selected bond distances in **sal** and **sal-1** to **sal-12** as well as the hypothetic conformer **sal-out**. See Sec. 4 of the main text for computational details. NMR isotopic shifts given in ppb, r_e values in Å, and $^n\Delta r$ values in μÅ, respectively.

		$^2\Delta$(C2)	r(O–$\underline{\text{H}}$)			r(O,O)			r(O$\cdots\underline{\text{H}}$)		
			r_e	$^n\Delta r_z$	$^n\Delta r_g$	r_e	$^n\Delta r_z$	$^n\Delta r_g$	r_e	$^n\Delta r_z$	$^n\Delta r_g$
**sal**	DD-VPT2	−205.52	0.9764	−991	−5993	2.6355	2530	2527	1.7688	2751	148
	loc-VPT2	−140.60		−1015	−6039		0	0		−99	−2637
	exp	−227									
**sal-1**	DD-VPT2	−120.24	0.9758	−275	−5309	2.6317	−1987	−1992	1.7723	−2709	−5314
	loc-VPT2	−126.62		−558	−5617		0	0		−96	−2636
	exp	−226									
**sal-2**	DD-VPT2	−598.14	0.9789	−4084	−8942	2.5654	14438	14434	1.6956	22871	20244
	loc-VPT2	−276.17		−3200	−8070		0	0		4383	1820
	exp	−303									
**sal-3a**	DD-VPT2	−278.63	0.9850	−2321	−6909	2.5766	4382	4378	1.6886	6717	4189
	loc-VPT2	−180.79		−2124	−6728		0	0		1491	−974
	exp	−304.00									
**sal-3b**	DD-VPT2	−334.26	0.9847	−2652	−7277	2.5891	7166	7163	1.7014	10995	8462
	loc-VPT2	−201.59		−2252	−6908		0	0		2212	−267
	exp	−332									
**sal-4**	DD-VPT2	−163.48	0.9796	−1132	−5930	2.6238	−1077	−1082	1.7472	−759	−3306
	loc-VPT2	−157.64		−1324	−6148		0	0		593	−1889
	exp	−245.00									
**sal-5**	DD-VPT2	−310.75	0.9934	−3571	−7899	2.5419	4946	4944	1.6357	8596	6106
	loc-VPT2	−210.36		−3185	−7540		0	0		2648	206
	exp	−420									
**sal-6**	DD-VPT2	−399.35	0.9798	−2821	−7594	2.5851	9224	9220	1.7066	14362	11772
	loc-VPT2	−216.98		−2383	−7183		0	0		2919	388
	exp	−290									
**sal-7**	DD-VPT2	−114.66	0.9819	−1042	−5784	2.6006	−635	−636	1.7237	−1181	−3725
	loc-VPT2	−136.43		−1299	−6066		0	0		84	−2404
	exp	−210									
**sal-8**	DD-VPT2	−198.97	0.9732	−761	−5940	2.6578	4537	4534	1.7940	5689	3029
	loc-VPT2	−136.44		−765	−5980		0	0		323	−2282
	exp	−170									
**sal-9**	DD-VPT2	−555.87	0.9832	−4333	−9027	2.5451	10182	10179	1.6684	18021	15430
	loc-VPT2	−285.44		−3520	−8222		0	0		4506	1988
	exp	−390									
**sal-10**	DD-VPT2	−596.21	0.9904	−4707	−9129	2.5151	9367	9364	1.6160	16467	13900
	loc-VPT2	−298.13		−3761	−8209		0	0		4461	1955
	exp	−640									
**sal-11**	DD-VPT2	−497.16	0.9960	−4559	−8963	2.4805	4328	4327	1.5790	12131	9556
	loc-VPT2	−301.37		−3946	−8359		0	0		5999	3465
	exp	−850									
**sal-12**	DD-VPT2	73.42	0.9778	842	−4286	2.6258	−9244	−9245	1.7675	−14907	−17532
	loc-VPT2	−69.74		16	−5114		0	0		−2499	−5073
	exp	−220									
**sal-out**	DD-VPT2	−71.71	0.9574	3615	−4879	2.7798	103	78			
	loc-VPT2	−62.65		3368	−5075		0	0			

Table S14: Isotope effects on selected mean-square amplitudes in **sal** and **sal-1** to **sal-12** as well as the hypothetic conformer **sal-out** for different methods. Calculations done at the ωB97X-D/pc-2 level of theory. See Sec. 4 of the main text for computational details. All values in mÅ^2 or dgr^2, respectively.

		r(O–H)		r(O,O)		r(O···H)		φ(C2–O–H)		τ(C1–C2–O–H)	
		all-H	IE	all-H	IE	all-H	IE	all-H	IE	all-H	IE
**sal**	DD-VPT2	5126	−1396	6433	−97.3	16292	−2801	46.6	−11.5	90.3	−24.7
	loc-VPT2	4957	−1450	0	0	7794	−2281	42.1	−12.3	80.9	−23.7
**sal-1**	DD-VPT2	5072	−1381	6141	−82.7	15771	−2779	46.7	−11.5	95.0	−25.1
	loc-VPT2	4906	−1435	0	0	7874	−2304	42.1	−12.3	81.8	−23.9
**sal-2**	DD-VPT2	5241	−1428	5438	−74.9	15007	−2792	46.2	−11.4	94.8	−23.8
	loc-VPT2	5063	−1481	0	0	7949	−2326	41.6	−12.2	76.9	−22.5
**sal-3a**	DD-VPT2	5428	−1479	5774	−86.0	15524	−2778	44.9	−11.0	87.0	−21.8
	loc-VPT2	5236	−1532	0	0	7791	−2280	40.4	−11.8	71.1	−20.8
**sal-3b**	DD-VPT2	5388	−1468	5812	−79.6	15304	−2718	44.7	−11.0	84.4	−22.1
	loc-VPT2	5199	−1521	0	0	7697	−2252	40.1	−11.7	72.4	−21.2
**sal-4**	DD-VPT2	5209	−1419	6117	−88.2	15705	−2746	45.9	−11.3	92.2	−23.3
	loc-VPT2	5036	−1474	0	0	7701	−2253	41.4	−12.1	76.5	−22.4
**sal-5**	DD-VPT2	5740	−1564	5297	−73.3	14835	−2739	43.3	−10.5	81.3	−20.0
	loc-VPT2	5522	−1616	0	0	7794	−2281	38.6	−11.3	76.9	−22.5
**sal-6**	DD-VPT2	5238	−1427	5538	−74.7	14704	−2689	45.4	−11.2	88.1	−23.2
	loc-VPT2	5062	−1481	0	0	7677	−2247	40.9	−12.0	76.0	−22.2
**sal-7**	DD-VPT2	5275	−1437	5739	−74.0	15175	−2731	45.7	−11.2	94.0	−23.0
	loc-VPT2	5098	−1492	0	0	7793	−2280	41.1	−12.0	75.1	−22.0
**sal-8**	DD-VPT2	4987	−1358	6063	−78.0	15063	−2654	46.8	−11.5	102.1	−26.4
	loc-VPT2	4829	−1413	0	0	7575	−2217	42.2	−12.4	86.5	−25.3
**sal-9**	DD-VPT2	5393	−1469	5358	−77.1	15277	−2856	45.7	−11.2	94.6	−22.6
	loc-VPT2	5200	−1522	0	0	8079	−2364	41.1	−12.0	72.5	−21.2
**sal-10**	DD-VPT2	5679	−1546	5070	−67.3	14601	−2754	43.9	−10.7	86.9	−20.8
	loc-VPT2	5459	−1597	0	0	7892	−2309	39.3	−11.5	67.2	−19.7
**sal-11**	DD-VPT2	5923	−1610	4840	−64.9	15049	−2919	43.9	−10.7	86.6	−20.2
	loc-VPT2	5674	−1660	0	0	8369	−2449	39.2	−11.5	65.3	−19.1
**sal-12**	DD-VPT2	5164	−1407	6380	−96.0	16886	−2947	47.4	−11.7	93.5	−25.2
	loc-VPT2	4991	−1461	0	0	8179	−2393	42.9	−12.5	82.3	−24.1
**sal-out**	DD-VPT2	4557	−1240	7985	−96.3			54.0	−13.6	181.7	−45.6
	loc-VPT2	4416	−1292	0	0			49.4	−14.5	167.5	−49.0

Table S15: Isotope effects on selected amplitude covariances with $r(\text{O}\underline{\text{H}})$ in **sal** and **sal-1** to **sal-12** as well as the hypothetic conformer **sal-out** for different methods. Calculations done at the ωB97X-D/pc-2 level of theory. See Sec. 4 of the main text for computational details. All values given in mÅ^2 or dgr^2, respectively.

		$r(\text{O,O})$		$r(\text{O}\cdots\underline{\text{H}})$		$\varphi(\text{C2–O–}\underline{\text{H}})$	
		all-H	IE	all-H	IE	all-H	IE
**sal**	DD-VPT2	14.0	99.3	−4626	1356	−44.4	7.2
	loc-VPT2	0	0	−4447	1301	−35.5	10.4
**sal-1**	DD-VPT2	77.3	93.1	−4456	1321	−43.8	7.0
	loc-VPT2	0	0	−4358	1275	−34.9	10.2
**sal-2**	DD-VPT2	37.0	98.7	−4752	1397	−50.5	8.9
	loc-VPT2	0	0	−4589	1343	−41.5	12.2
**sal-3a**	DD-VPT2	−52.4	108.1	−5109	1479	−51.7	9.3
	loc-VPT2	0	0	−4826	1412	−42.9	12.5
**sal-3b**	DD-VPT2	−29.3	103.9	−5020	1457	−48.9	8.5
	loc-VPT2	0	0	−4771	1396	−40.1	11.7
**sal-4**	DD-VPT2	18.2	100.0	−4746	1392	−45.5	7.5
	loc-VPT2	0	0	−4567	1336	−36.7	10.7
**sal-5**	DD-VPT2	−101.1	114.0	−5527	1586	−55.0	10.2
	loc-VPT2	0	0	−5164	1511	−46.4	13.6
**sal-6**	DD-VPT2	55.7	97.4	−4742	1399	−46.5	7.8
	loc-VPT2	0	0	−4604	1347	−37.8	11.1
**sal-7**	DD-VPT2	30.4	98.1	−4778	1402	−46.2	7.7
	loc-VPT2	0	0	−4613	1350	−37.5	11.0
**sal-8**	DD-VPT2	131.4	88.7	−4311	1294	−37.8	5.3
	loc-VPT2	0	0	−4285	1254	−29.2	8.5
**sal-9**	DD-VPT2	−16.0	104.9	−5017	1460	−56.1	10.5
	loc-VPT2	0	0	−4771	1396	−47.0	13.7
**sal-10**	DD-VPT2	−74.2	110.2	−5428	1561	−56.5	10.6
	loc-VPT2	0	0	−5089	1489	−47.6	13.9
**sal-11**	DD-VPT2	−115.9	116.3	−5792	1651	−69.8	14.2
	loc-VPT2	0	0	−5364	1570	−59.6	17.4
**sal-12**	DD-VPT2	−9.2	100.7	−4688	1367	−50.6	9.0
	loc-VPT2	0	0	−4473	1309	−41.2	12.1
**sal-out**	DD-VPT2	−272.6	−79.7			−21.2	0.6
	loc-VPT2	0	0			−12.4	3.6

Table S16: Equilibrium values for and isotope effects on selected bond angles in **sal** and **sal-1** to **sal-12** as well as the hypothetic conformer **sal-out**. See Sec.4 of the paper for computational details. Equilibrium angles given in dgr, isotope effects in mdgr.

	φ(C2–O–H)			φ(C1–C2–O)		φ(C1–C7=O)		φ(C2–C1–C7)	
	φ_e	$^n\Delta\varphi_z$		φ_e	$^n\Delta\varphi_z$	φ_e	$^n\Delta\varphi_z$	φ_e	$^n\Delta\varphi_z$
		DD-VPT2	loc-VPT2		DD-VPT2		DD-VPT2		DD-VPT2
**sal**	107.865	−88	−99	121.877	4	124.504	28	120.483	51
**sal-1**	107.706	−61	−57	123.553	−36	124.348	−12	119.544	−24
**sal-2**	107.370	240	181	124.277	231	124.269	87	117.255	179
**sal-3a**	107.727	−16	−34	120.822	21	123.270	47	120.204	78
**sal-3b**	107.378	61	33	121.098	58	124.219	72	120.122	116
**sal-4**	108.073	−67	−56	120.675	9	124.336	−33	120.779	−20
**sal-5**	107.279	4	−11	120.206	16	123.073	59	119.970	89
**sal-6**	107.625	126	99	121.805	125	124.559	69	119.087	125
**sal-7**	108.057	−112	−106	121.798	−51	123.606	6	119.709	11
**sal-8**	108.269	−15	−33	120.899	41	124.660	43	121.036	73
**sal-9**	107.171	208	166	124.360	207	123.974	28	117.191	115
**sal-10**	107.023	171	139	122.295	112	124.365	81	117.698	128
**sal-11**	106.222	297	288	123.781	105	122.914	−1	115.982	25
**sal-12**	107.501	−310	−251	123.685	−163	124.116	−49	119.490	−113
**sal-out**	109.702	−223	−254	119.116	11	127.294	−2	124.356	0

References

S1 Schaftenaar, G.; Noordik, J.H. Molden: a pre- and post-processing program for molecular and electronic structures. *J. Comp.-Aided Mol. Design* **2000**, *14*, 123–134. doi:10.1023/A:1008193805436.

S2 Gräfenstein, J. Efficient calculation of NMR isotopic shifts: Difference-dedicated vibrational perturbation theory. *J. Chem. Phys.* **2019**, *151*, 244120. doi:10.1063/1.5134538.

S3 Berger, S.; Diehl, B.W.K. Correlation between deuterium-isotope effects and ^{13}C-NMR chemical-shifts in substituted benzenes. *Tetrahedron Lett.* **1987**, *28*, 1243–1246. doi:10.1016/S0040-4039(00)95336-X.

S4 Yang, K.S.; Hudson, B. Computation of deuterium isotope perturbation of ^{13}C NMR chemical shifts of alkanes: A Local Mode Zero-Point Level approach. *J. Phys. Chem. A* **2010**, *114*, 12283–12290. doi:10.1021/jp105913x.

S5 Aydin, R.; Wesener, J.R.; Günther, H.; Santillan, R.L.; Garibay, M.E.; Joseph-Nathan, P. On the stereospecifity of intrinsic ^{2}H–^{1}H NMR isotope effects on carbon-13 chemical-shifts in cyclohexanes. *J. Org. Chem.* **1984**, *49*, 3845–3847. doi:10.1021/jo00194a040.

S6 O'Leary, D.J.; Allis, D.G.; Hudson, B.S.; James, S.; Morgera, K.B.; Baldwin, J.E. Vicinal deuterium perturbations on hydrogen NMR chemical shifts in cyclohexanes. *J. Am. Chem. Soc.* **2008**, *130*, 13659–13663. doi:10.1021/ja802903a.

S7 Aydin, R.; Frankmölle, W.; Schmalz, D.; Günther, H. Deuterium-Induced NMR Isotope Shifts for ^{13}C Resonance Frequencies of Norbornane - Quantitative Data for the Dihedral Angle Dependence of Vicinal Shifts. *Magn. Res. Chem.* **1988**, *26*, 408–411. doi:10.1002/mrc.1260260511.

S8 Mlinarić-Majerski, K.; Vinković, V.; Meić, Z.; Gassman, P.G.; Chyall, L.J. Long-Range Deuterium-Isotope Effects In ^{13}C NMR-Spectra Of Adamantane And 2-Adamantanone. *J. Mol. Struct.* **1992**, *267*, 389–394. doi:10.1016/0022-2860(92)87061-Y.
